# 3-T MRI *in vitro* characterisation of a SPION-polymer balloon catheter: a proof-of-concept study

**DOI:** 10.1186/s41747-026-00778-z

**Published:** 2026-08-13

**Authors:** Paul E. Hermann, Thomas Friedrich, Mandy Ahlborg, Maren Friederike Balks, Amelie Wüllner, Wyger M. Brink, Maria-Josephina Buhné, Martin A. Koch, Malte M. Sieren, Dennis Kundrat, Thorsten M. Buzug, Roman Kloeckner, Jörg Barkhausen, Alex Frydrychowicz, Franz Wegner

**Affiliations:** 1 Institute of Interventional Radiology, University of Lübeck, Lübeck, Germany; 2 Fraunhofer IMTE, Fraunhofer Research Institution for Individualized Medical Technology and Engineering, Lübeck, Germany; 3 Section of Pediatric Radiology, Department of Diagnostic and Interventional Radiology and Nuclear Medicine, University Medical Center Hamburg-Eppendorf, Hamburg, Germany; 4 Institute of Radiology and Nuclear Medicine, University of Lübeck, Lübeck, Germany; 5 Department of Magnetic Detection and Imaging, TechMed Centre, University of Twente, Enschede, The Netherlands; 6 Institute of Medical Engineering, University of Lübeck, Lübeck, Germany

**Keywords:** Angioplasty (balloon), Catheters, Endovascular procedures, Magnetic resonance imaging (interventional), Nanoparticles

## Abstract

**Objective:**

Magnetic resonance imaging (MRI)-guided endovascular interventions represent a radiation-free alternative to x-ray fluoroscopy but remain limited by the lack of compatible and visible instruments. This study aimed to characterise a balloon catheter with polymer-embedded superparamagnetic iron oxide nanoparticles (SPIONs) as a passive MR-visible marker.

**Materials and methods:**

The SPION balloons consisted of a polymer with integrated iron oxide particles. A custom-built phantom study was conducted on a clinical 3-T MRI scanner using a balanced steady-state free precession sequence. Parameters influencing the balloons’ imaging properties were evaluated: SPION content (5 weight percent (wt%), 20 wt% and 30 wt%), flip angle (10° to 60°), the balloons’ spatial orientation relative to the main magnetic field B_0_, the inflation state and phase-encoding direction across different spatial orientations. The SPION-induced susceptibility artefacts were quantified from segmentations independently performed by two board-certified radiologists.

**Results:**

All SPION-polymer balloons were discernible at 3-T MRI across all tested SPION concentrations. No linear correlation was observed between SPION concentration and artefact dimension, with the intermediate concentration of 20 wt% yielding the smallest artefact size. The balloons’ artefact dimensions increased with higher angulations relative to B_0_. Balloons were visualised in the inflated and deflated configurations. The flip angle had a limited impact on artefact dimensions, while higher flip angles resulted in increased SNR. The influence of phase-encoding direction was generally minor with a single exception (90° coronal plane, AP to RL).

**Conclusion:**

The SPION-polymer balloons were effectively visualised at 3-T MRI irrespective of orientation and inflation. This *in vitro* study outlines a proof-of-concept supporting future clinical use of polymer-integrated SPIONs for passive device visualisation in interventional MRI.

**Relevance statement:**

SPION-polymer balloons can be visualised in 3-T MRI, providing a foundation for future MRI-visible interventional instrument designs.

**Key Points:**

- Visualisation properties of a SPION-polymer balloon catheter were systematically assessed at 3-T MRI to overcome the shortage of MRI-visible instruments.
- SPION-polymer balloons were effectively visualised across different spatial orientations in the inflated and deflated state.
- Polymer-integrated SPIONs demonstrate potential as passive instrument markers for MRI-guided interventions.

**Graphical Abstract:**

**Figure:**
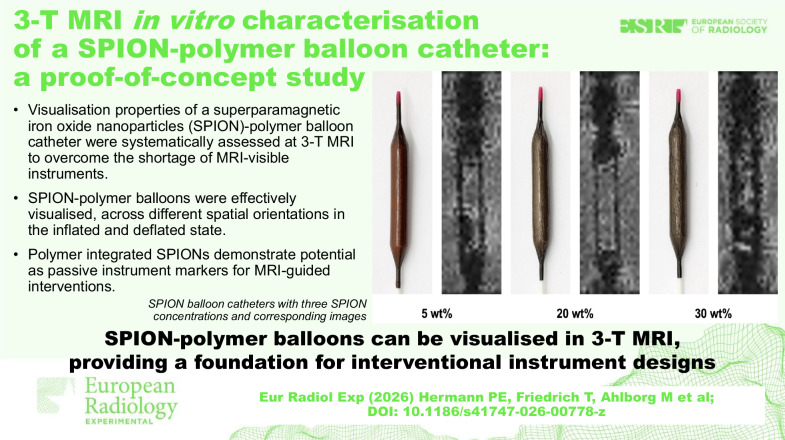

## Background

Magnetic resonance imaging (MRI) represents a radiation-free alternative to conventional x-ray fluoroscopy for instrument guidance during vascular interventions. The potential of interventional MRI has been demonstrated by numerous animal studies and reported human applications [1–4]. The advantages of MRI-guided procedures over x-ray-based techniques include simultaneous excellent soft-tissue contrast, multiplanar reconstruction properties, functional imaging capabilities and independence from iodine contrast agents. The potential of MRI-guided interventions is further strengthened by emerging deep learning algorithms for device tracking and automated slice selection [5–8].

Apart from scanner availability, one fundamental limitation to a broader clinical implementation is the sparsity of MRI-compatible instruments. In addition to safety issues, sufficient and reliable visualisation in MRI remains challenging [9]. Conventional endovascular catheters made of polymers are not susceptible to MR-signal generation. The resulting signal voids may be too small to reliably monitor the catheter during an endovascular intervention.

For balloon catheters, which are commonly used for percutaneous angioplasty or stent deployment, various approaches for MR visualisation have been proposed. Fundamentally, active visualisation methods can be distinguished from passive techniques.

In active methods, either receiver coils, antennas or current-controlled markers are attached to the device and induce a signal in the MR-receive coils [10]. The MRI scanner’s software and hardware may need to fulfil special requirements for these applications, and additional components are necessary. Especially for balloon catheters, it can be technically challenging to incorporate active elements onto the balloon surface due to biomechanical properties and the deformation process during inflation and deflation. Moreover, RF-pulse-induced heating of electrically conductive coils and antennas represents a major safety concern [11, 12].

Passive labelling methods usually do not require dedicated technical prerequisites and can be divided into negative and positive contrast principles. Filling a balloon with air for negative contrast can lead to its visualisation in MRI. However, the resulting signal void may be indistinguishable from incidental signal void artefacts [13] or susceptibility-induced artefacts, potentially leading to misinterpretation of balloon size and adjacent structures. Moreover, this method is associated with the risk of air embolism in the event of balloon rupture [14]. Filling the balloon with a contrast agent, such as a gadolinium-doped solution for positive contrast, limits the choice of sequence types [1]. Most importantly, visualisation is restricted to the inflated state. Thus, the MR visualisation during the positioning of a deflated balloon catheter is not supported by these methods. Modifying the balloon by applying contrast markers on the surface enables visualisation of the balloon catheter irrespective of its configuration [15]. The main disadvantage of this method is that these markers may impair biocompatibility, *e.g*., due to possible prothrombogenic effects or marker fragmentation/dislodgement.

The integration of superparamagnetic iron oxide nanoparticles (SPIONs) as passive negative contrast markers into the catheter polymer may overcome the disadvantages of existing marking approaches. SPIONs have proven effective as passive negative markers due to resulting susceptibility artefacts in MRI [15–17]. In addition to their biocompatibility, embedding SPIONs into the polymer preserves the balloon’s smooth, biomechanical surface properties and may allow visualisation regardless of the balloon’s inflation state. Moreover, the consistent SPION-induced negative contrast pattern may facilitate deep learning-based device tracking. However, the imaging performance and visualisation properties of balloon catheters with polymer-integrated SPIONs in MRI have not yet been systematically characterised.

Hence, the aim of this study was to test the hypothesis that incorporating SPIONs into the balloon polymer enables stable MR visualisation by producing a balanced amount of susceptibility artefacts.

## Methods

### Balloon catheters

The evaluated SPION-polymer balloon catheters were originally developed and tested for visualisation in Magnetic Particle Imaging (MPI) [18]. However, the SPIONs used are not limited to MPI. The concept of SPIONs as contrast agents and instrument markers in MRI, as well as their potential as bimodal MPI/MRI signal contributors have already been established elsewhere [17, 19, 20]. The balloon polymer consists of two components: MagSilica Type 1 and VESTAMID Care ML21 (Evonik Industries AG, Essen, Germany). MagSilica Type 1 is a particle with an iron oxide (Fe_2_O_3_) core embedded in a closed silica (SiO_2_) coating, facilitating integration into polymers. VESTAMID Care ML21 is a biocompatible long-chained polyamide suited for medical purposes. These two components were merged *via* a dry blend procedure. Three versions of the polymer containing 5 weight percent (wt%), 20 wt% and 30 wt% SPIONs were compounded. The balloons were manufactured from these three different polymers from stretched tubes using a moulding process. Laser welding was used to connect the balloon to the catheter shaft. Investigations regarding the safety in medical applications had been conducted in prior work: The balloon granulate was tested as non-haemolytic and non-cytotoxic [18]. The nominal pressure of the balloons was 3.5 bar and the burst pressure 7.23 bar. The dimensions of the balloons were 6 mm in diameter and 20 mm in length. The balloon catheters were inflated with 0.9% saline solution during the conducted experiments. To establish an inflated and crimped configuration for the imaging studies, the balloons were first evacuated and flattened out, then crimped in a rolled-up configuration into the sleeve of a conventional 6 mm angioplasty balloon. Except for the SPION-concentration study, all subsequent studies were conducted with a 20 wt% balloon. This intermediate SPION concentration was selected as it was expected to provide an optimal balance between mechanical properties (*e.g*., burst pressure) and anticipated MRI visibility.

### Experimental setup

**Fig. 1:**
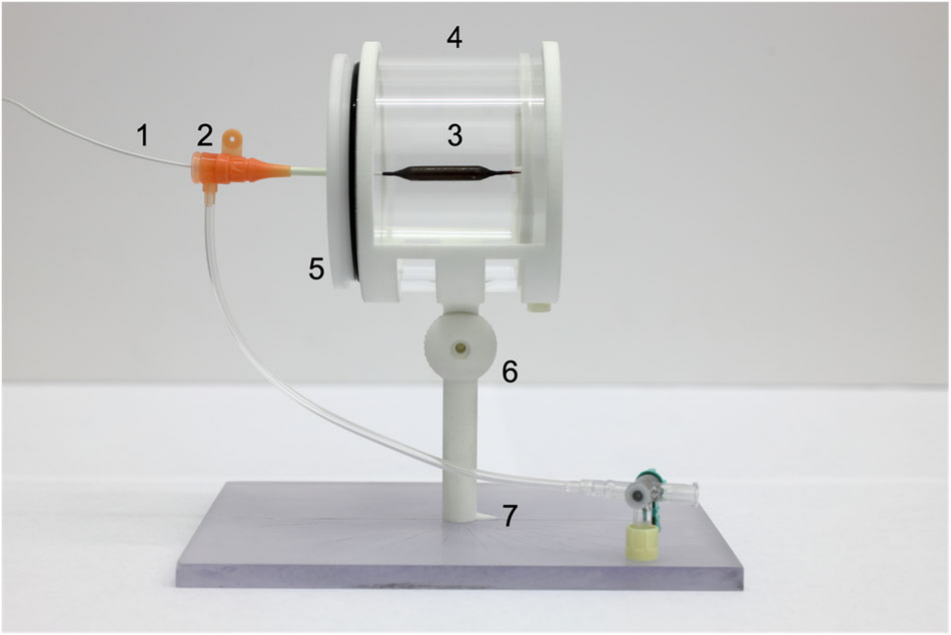
Overview of the experimental setup: Angle-adjustable phantom loaded with a 20 wt% SPION balloon *via* 7 French vascular introducer sheath. (1) SPION balloon catheter (shaft), (2) shortened vascular introducer sheath, (3) SPION balloon catheter (balloon), (4) acrylic glass chamber and 3D-printed mount, (5) closing lid with O-ring seal, (6) hinge joint, (7) swivel joint between mount and base plate

For the imaging studies, a custom-made phantom with an angle-adjustable mount was produced. This phantom allowed the alignment of the balloons in any spatial orientation relative to the main magnetic field B_0_ in 10° increments. The chamber containing a single balloon was produced from acrylic glass, and a removable lid was manufactured from polyoxymethylene (Fig. 1). Both materials were selected because their magnetic susceptibility is close to that of water and therefore they have a low propensity to cause MRI artefacts [21]. For fluid-tight sealing, a rubber O-ring was installed between the chamber and lid. To access the chamber with the balloon catheter, a manually shortened commercial vascular introducer sheath (7 French, Radifocus II, Terumo, Shibuya, Japan) was installed into the lid. The chamber had an inner diameter of 73 mm, an inner length of 55 mm and a wall thickness of 3.4 mm. The chamber size was chosen to be larger than a human vessel with the intention to generate a homogenous surrounding and thus ideal conditions for the artefact quantification. The chamber was connected to an acrylic glass base plate by a 3D-printed polyamide mount. This mount had a hinge joint and was freely rotatable on the base plate through a swivel joint, allowing adjustment to different angles around two axes.

For the imaging experiments, the phantom chamber was filled with gadolinium-doped saline solution (1:200). The balloon-loaded phantom was placed in the isocentre of the scanner. A box (polypropylene) was placed above the phantom to hold the receiver coil.

As the MR-visible volume of the phantom alone was insufficient to ensure imaging sequence initiation, two 1-litre bottles of aqueous copper sulphate (CuSO_4_) solution (Phantom Bottle 1000cc L11, Philips Healthcare, Hamburg, Germany) were placed adjacent to the phantom, perpendicular to the B_0_ field and at approximately 15 cm distance to avoid any interference. The reporting of this phantom study was, wherever possible, aligned to the Phantom Studies in Medical Imaging (PSMI) checklist and guidelines.

### Evaluated parameters

To evaluate the balloons’ visualisation characteristics, five different parameters were investigated: (1) The influence of SPION concentration within the polymer; (2) The balloon configuration in an inflated state compared to a deflated and crimped state; (3) The balloons’ spatial orientation relative to the main magnetic field B_0_, with angles of 0° (parallel to B_0_), 30°, 60° and 90° (perpendicular to B_0_) in the coronal (xz) and sagittal (yz) planes; (4) The influence of different flip angles (FA) from 10° to 60° in 10° increments; (5) The influence of the phase-encoding direction in parallel and perpendicular alignment to B_0_ in the coronal and sagittal planes. For every investigated condition, one single measurement with the same balloon catheter was carried out.

### MRI setup

The MR images were acquired on a clinical 3-T scanner (Magnetom Vida, Siemens, Erlangen, Germany), using an 18-channel phased-array body coil (Body 18, Siemens, Erlangen, Germany). A real-time capable balanced steady-state free precession (bSSFP) sequence was used. The following parameters were applied: TR/TE = 5.04/2.52 ms; Matrix = 128 × 128; FOV = 100 × 100 mm, slice thickness = 1.5 mm; band width = 558 Hz/pixel. The default phase-encoding direction was anterior-posterior (AP) direction and was only changed when assessing the effect of phase-encoding direction. A default FA of 40° was used and only varied for the FA experiments. The parameters were chosen for their compatibility with real-time cine bSSFP imaging.

### Image analysis

**Fig. 2:**
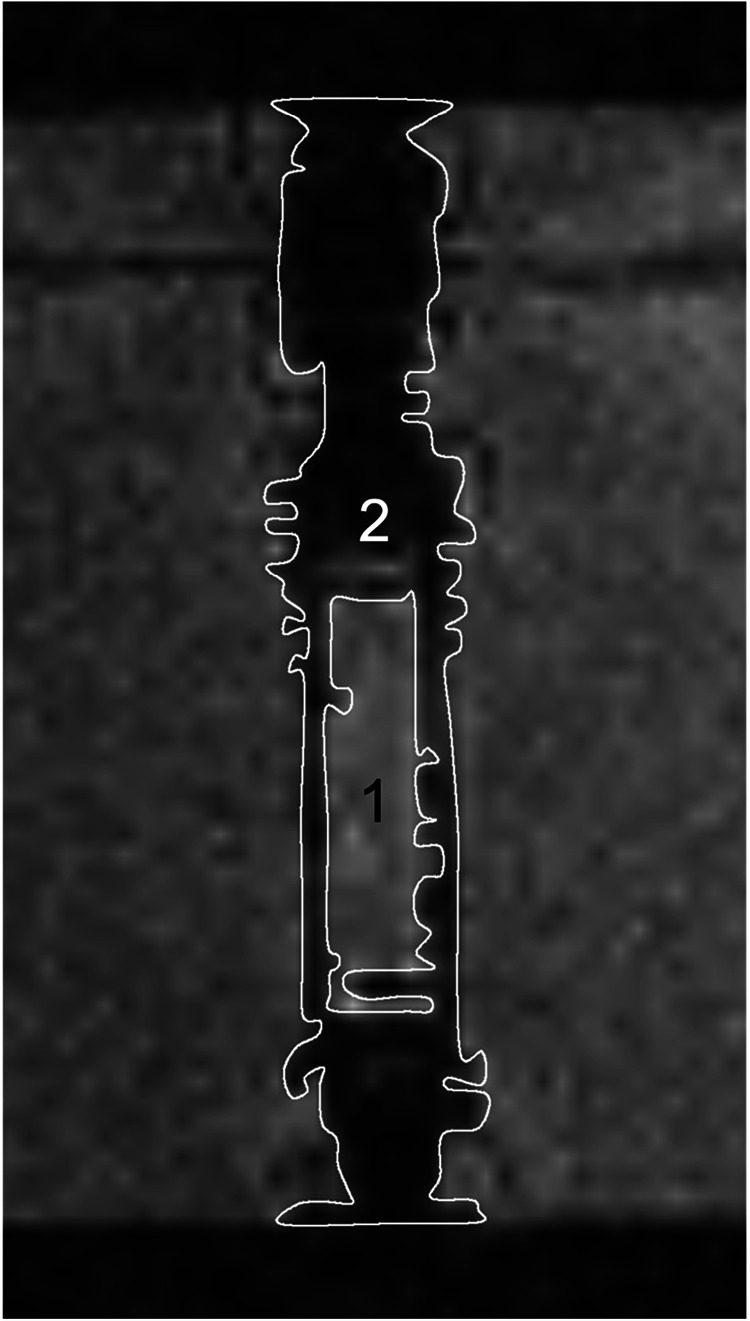
Representative segmentation of a balloon’s artefact dimensions. The luminal area (1) was subtracted from the measured outer dimensions (2) to calculate the net artefact area

To quantify the susceptibility artefact dimensions, all images were independently and blindly segmented on a clinical workstation by two board-certified radiologists with 5 years (M.J.B.) and 9 years (F.W.) of experience (Suppl. Table 1). Segmentation was performed using the measurement tool of the *DeepUnity Diagnost* PACS viewer (Version 1.2.0.3, Dedalus S.p.A., Milan, Italy). The rationale for the chosen manual segmentation approach was to represent the physician’s subjective discernibility, which is most relevant under clinical reading conditions. For each dataset, the central slice was preselected before segmentation, with the catheter shaft usually being visible within the inflated balloon’s lumen. The central slice was chosen because the artefact dimensions were largest there and in an anticipated clinical scenario, *e.g*., stenosis would be most prominent there. First, the artefacts’ outer contours were delineated. Second, the artefacts’ inner contours were marked in case of inflated balloons. For analysis, the net artefact area was defined as the difference between the outer and inner contour area (Fig. 2).

For the FA study, signal-to-noise ratio (SNR) approximations for a comparative analysis were carried out. To measure the signal intensity, the largest possible circular region of interest (ROI) was placed in the centre of the inner contour without overlapping the balloon artefacts. To approximate noise, a second circular ROI with a diameter of 10 mm was placed on the midline at the edge of the FOV, at the maximum possible distance from the balloon artefacts. Mean values were calculated from the measurement results of both raters. SNR values were intended to demonstrate general trends within this substudy, given the inhomogeneous noise distribution inherent to phased-array coils.

### Interrater reliability

To evaluate the reliability of the measurements performed by the two raters, an intraclass correlation coefficient (ICC) was calculated using MATLAB (R2025b, The MathWorks, Inc., Natick, MA, USA). The two-way random, single score (ICC (2,1)) agreement model was chosen. To interpret the ICC-scores, the following intervals were used: ICC < 0.50 poor, ICC ≥ 0.50 to < 0.75 moderate, ICC ≥ 0.75 to < 0.90 good and ICC ≥ 0.90 excellent reliability [22].

## Results

### SPION concentration

**Fig. 3:**
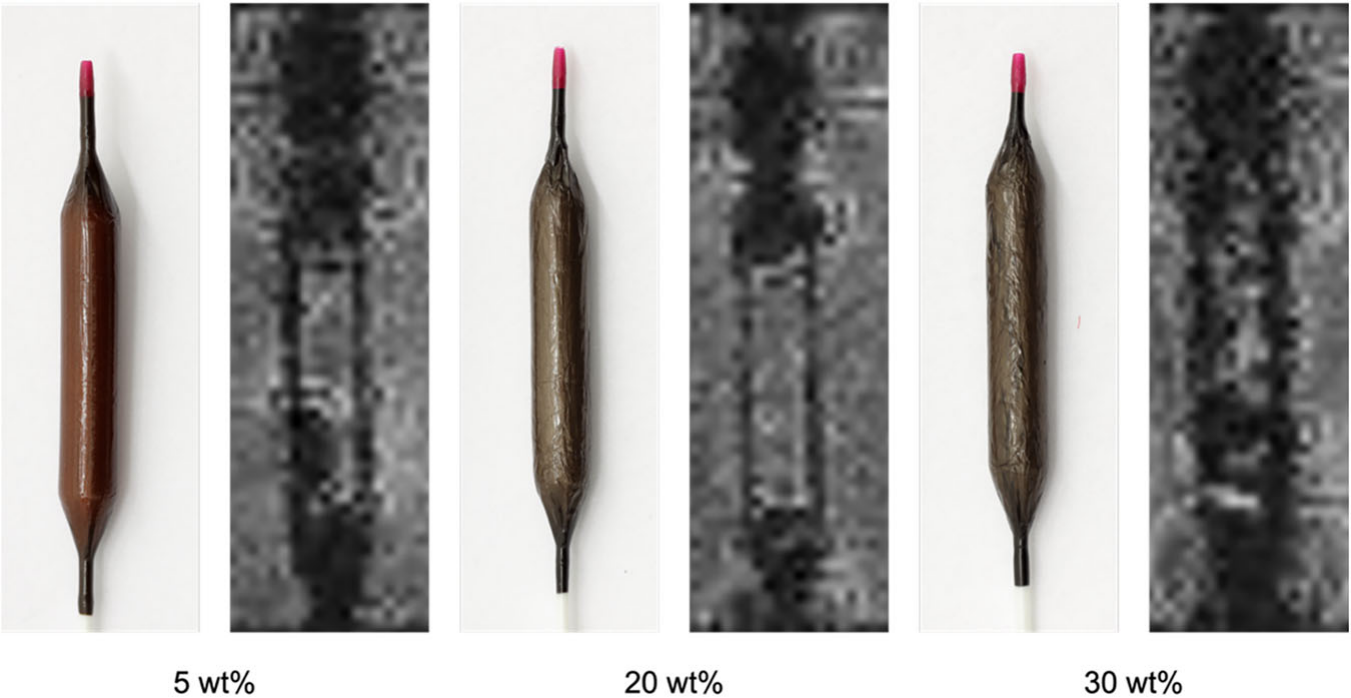
Photographs of balloon catheters with three SPION concentrations (5 wt%, 20 wt% and 30 wt%) with corresponding MR images. The greyish-brown colour of the balloons is caused by the iron oxide particles. A colour gradient with rising SPION concentrations towards darker brown tones is observed

All three versions of the balloon containing 5 wt%, 20 wt% and 30 wt% SPIONs exhibited pronounced susceptibility artefacts. For all versions, the outer contour as well as the inner contour, delimiting the balloon’s lumen, were clearly distinguishable in the inflated state (Fig. 3). The highest SPION concentration resulted in the largest artefact dimensions (mean: 310.4 mm^2^, Rater A: 342.4 mm^2^, Rater B: 278.3 mm^2^). The 20 wt% variant exhibited the smallest artefact dimensions (mean: 214.2 mm^2^, Rater A: 235.0 mm^2^, Rater B: 193.5 mm^2^). The 5 wt% variant exhibited intermediate artefact dimensions (mean: 253.3 mm^2^, Rater A: 261.8 mm^2^, Rater B: 244.8 mm^2^).

### Flip angle

**Fig. 4:**
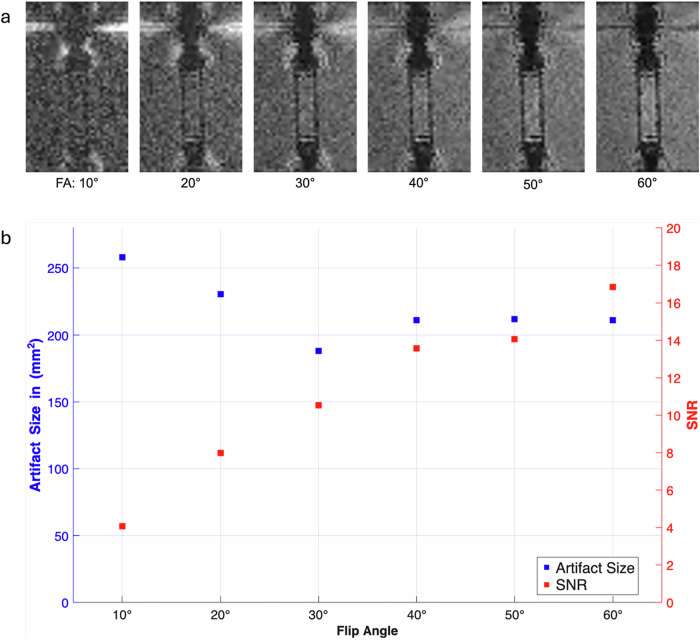
**a** MR images of the balloon catheter acquired with varying flip angles. **b** Scatter plot of artefact dimensions (blue) and SNR (red) for FAs ranging between 10° and 60°. The SNR was strongly dependent on FA, whereas artefact dimensions showed only a slight decrease with increasing FA

The artefact dimensions were not appreciably influenced by different FA settings. Only a modest decrease in artefact dimensions with increasing FA could be observed (257.9 mm^2^ at FA = 10° to 211.2 mm^2^ at FA = 60°). In contrast, the SNR of the images was markedly influenced by the chosen FA (Fig. 4 and Suppl. Table 2). With gradually increasing FA, the SNR increased accordingly (SNR = 4.1 at FA = 10° to SNR = 16.8 at FA = 60°).

### Spatial orientation of the balloon catheter

**Fig. 5:**
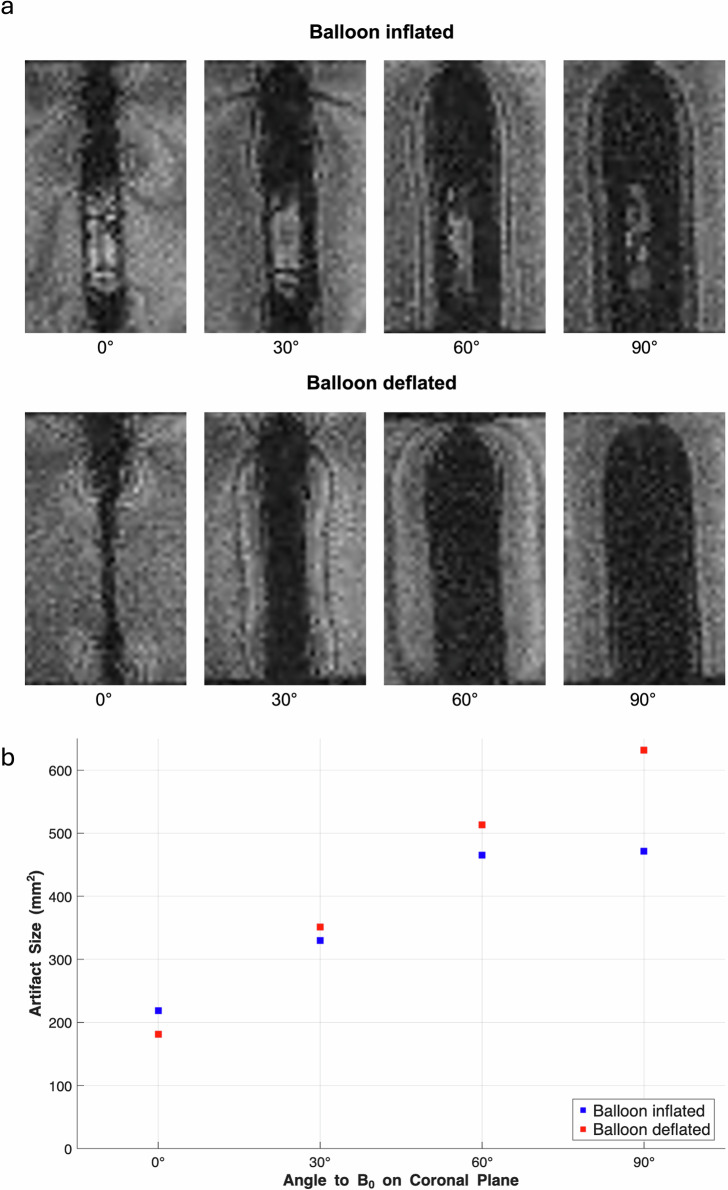
**a** MR images of the balloon catheter in different spatial orientations indicated as angles relative to B_0_ on the coronal (xz) plane, in the inflated state (upper row) and in the deflated state (lower row). **b** Scatter plot of artefact dimensions for different orientations for the inflated and deflated balloon. The artefact dimensions increased gradually with increasing angles relative to B_0_ for both the inflated and deflated states. For the inflated state, the increase was less pronounced for higher angulations (60° and 90°)

**Fig. 6:**
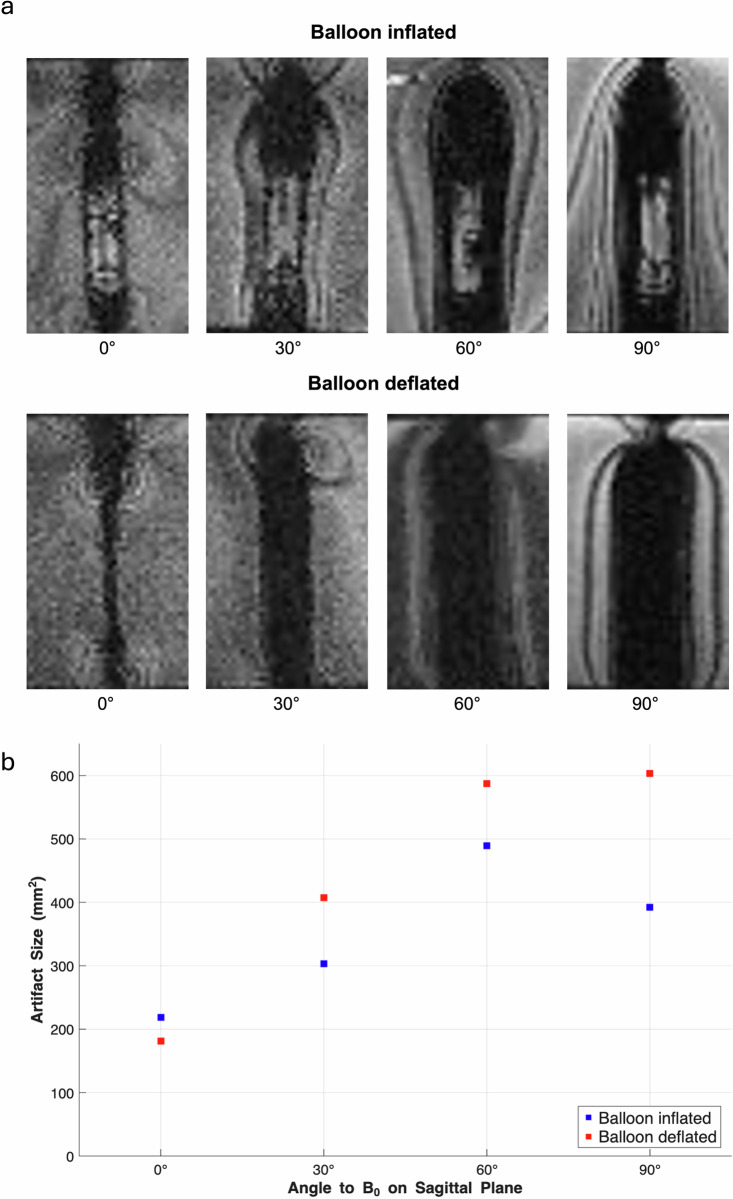
**a** MR images of the balloon catheter in different spatial orientations indicated as angles relative to B_0_ on the sagittal (yz) plane, in the inflated state (upper row) and in the deflated state (lower row). **b** Scatter plot of artefact dimensions for different orientations for the inflated and deflated balloon. Similar effects to those observed on the coronal plane are shown here, with less distinctive changes at higher angulations relative to B_0_ (60° and 90°)

The balloons’ spatial orientation had a major influence on the resulting artefact dimensions. With increasing angles from 0° to 90° relative to the B_0_ field, the artefact dimensions increased consistently, both in the coronal (Fig. 5) and in the sagittal plane (Fig. 6). This trend was observed in both configurations of the balloon—inflated and deflated. For the inflated balloon, this corresponded to a fold-change of 2.15 in the coronal plane and 1.79 in the sagittal plane at 90° relative to the 0° baseline.

With increasing angles to B_0_, these effects were less distinctive, especially in the inflated state. Only a small increase in artefact dimensions could be observed when comparing the 60° to the 90° position in the coronal plane (from 465.1 mm^2^ to 471.5 mm^2^). Notably, in the sagittal plane, there was even a reduction in artefact dimensions between the 60° and 90° position (from 488.9 mm^2^ to 391.9 mm^2^).

### Balloon configuration

In both the inflated and deflated (crimped) configurations, the balloons exhibited substantial susceptibility artefacts and were clearly distinguishable. The deflated, crimped configuration, as expected, showed no visible lumen. The overall trend of increased artefact dimensions with greater angulations relative to B_0_ was broadly similar between both inflation states. Notably, the deflated balloon exhibited larger artefacts at angulated positions compared to the inflated balloon.

### Phase-encoding direction

The phase-encoding direction generally had only a small effect on the artefact dimensions across the tested spatial orientations. However, a subtle trend could be observed: a phase-encoding direction set to AP generally resulted in smaller artefact dimensions compared to head-feet (HF) and right-left (RL) directions (*e.g*., 0° position relative to B_0_: 202.9 mm^2^ for AP and 217.3 mm^2^ for HF).

**Fig. 7:**
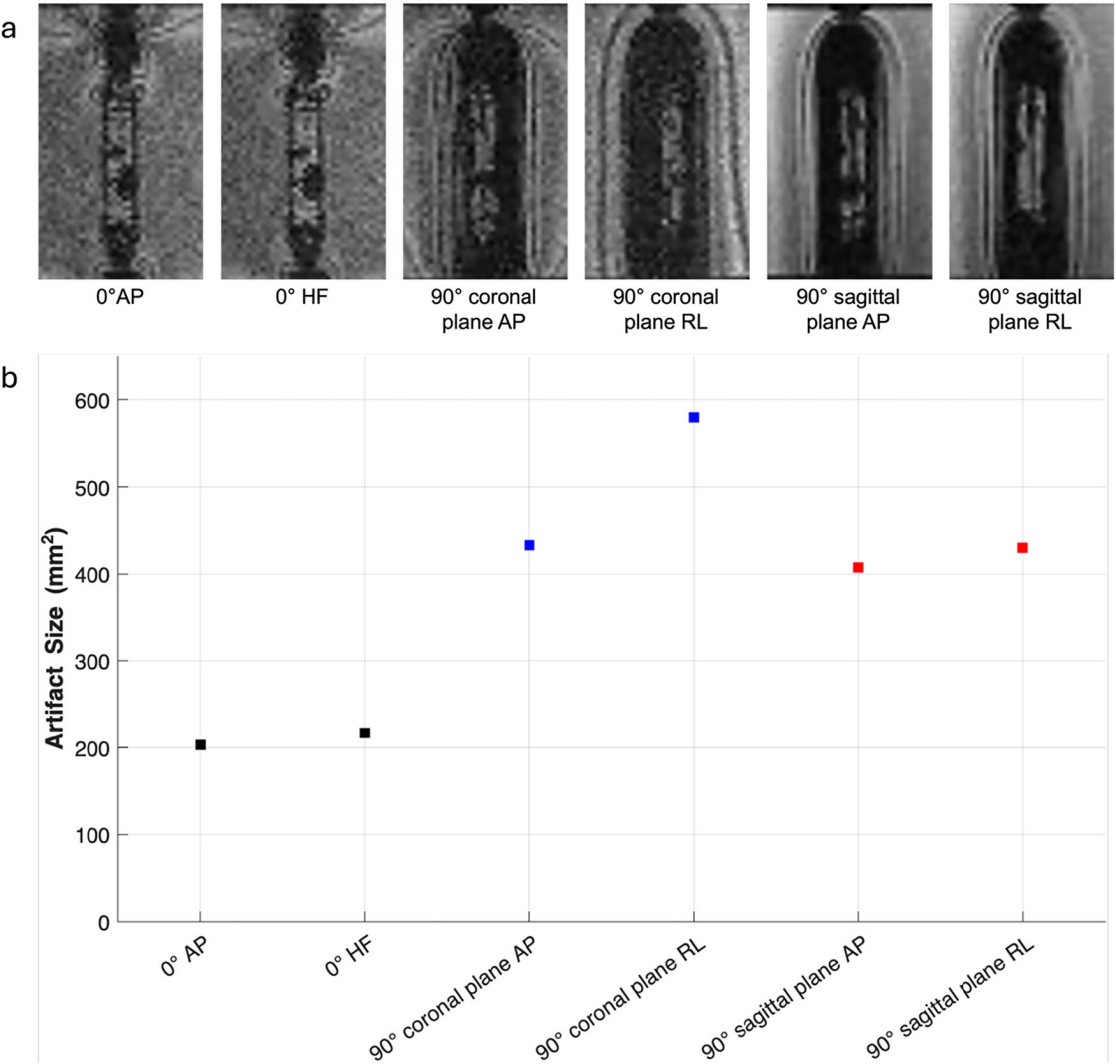
**a** MR images acquired with different phase-encoding directions and spatial orientations as indicated. **b** Scatter plot of artefact dimensions for the respective combinations of phase-encoding directions and spatial orientations. A phase-encoding direction set to anterior-posterior (AP) generally led to slightly smaller artefact dimensions compared to head-feet (HF) and right-left (RL) directions. One considerable exception from this trend was observed at 90° in combination with right-left phase-encoding direction on the coronal plane

The 90° position in the coronal plane showed a notable deviation from this trend (Fig. 7). Switching the phase-encoding direction from AP to RL resulted in a considerably larger increase in artefact dimensions here (432.7 mm^2^ for AP, 579.2 mm^2^ for RL).

### Interrater reliability

The ICC was calculated for artefact area segmentations across all conducted studies, resulting in a value of 0.904 (95% CI: 0.885, 0.913). According to the predefined thresholds, an ICC ≥ 0.90 was interpreted as indicating excellent interrater reliability.

## Discussion

This work is the first to systematically characterise the imaging properties of a SPION-polymer balloon catheter for interventional MRI in a proof-of-concept study. The evaluated SPION balloons demonstrated favourable visualisation characteristics in 3-T MRI using a bSSFP sequence under various parameter conditions. Therefore, the integration of SPIONs into the device’s polymer represents a promising approach for the targeted MR visualisation of non-metallic instruments.

The balloon catheters were discernible across all tested FAs between 10° and 60°, although with lower FAs, particularly 10° and 20°, the balloons may not be sufficiently visualised for clinical conditions due to very low SNR values. The artefact dimensions showed no increase with increasing FA up to 60° in this work. In a previous investigation of needle artefacts in MRI, artefact dimensions were constant up to 60° but increased when applying FAs larger than 60° [23]. It should be acknowledged that the use of higher FAs, generally leading to superior SNRs, may be limited for safety reasons, particularly due to an increase in the specific absorption rate (SAR) [24]. Hence, FAs around 40° appear to provide a good trade-off between artefact dimensions and SAR considerations.

Surprisingly, the different SPION concentrations of the balloons showed no linear effect on the resulting artefact dimensions in MRI. Although the highest SPION concentration (30 wt%) led to the largest artefact dimension, the intermediate SPION concentration (20 wt%) showed smaller artefacts than the low SPION concentration (5 wt%). A potential explanation for this phenomenon could be the formation of SPION clusters within the polymer matrix. Former micro-CT analyses of the tested balloons revealed SPION agglomerates up to 30 µm in size [18]. Although the physical principles of image generation differ between MRI and MPI, SPION agglomeration has also been associated with a nonlinear increase in MPI signal intensity with increasing SPION proportion. However, the dependency between SPION concentration and the amount of agglomeration remains unclear.

Notably, a native balloon of the identical type without radiopaque markers would have enabled a more robust quantification of the SPION-enhanced visibility, as the polyamide may also have contributed to the negative contrast. Unfortunately, such a balloon was unavailable.

Our systematic approach revealed the spatial orientation of the balloon catheters to have the most pronounced influence on the resulting artefact dimensions in this study. Similar effects of increasing susceptibility artefacts with increasing deviation from parallel alignment to B_0_ have been reported for metallic stents [25] and biopsy needles in MRI [23]. Nevertheless, the observed decrease in artefact size for the 60° to 90° angle in the sagittal plane represents an exception to this general trend. A partial volume effect may offer a plausible explanation, though the underlying mechanism remains uncertain at present.

With increasing artefact dimensions, the potential risk of obscuring anatomical structures or pathologies, such as the targeted stenosis, increases. Additionally, changing the visualisation characteristics of the balloon throughout an interventional course could be challenging and must be anticipated by the operating physician. This is highly relevant in vasculatures with large angle deviations, *e.g.*, in the treatment of renal artery stenoses. During this procedure, when the balloon is advanced from the aorta into a renal artery—which originates at a nearly perpendicular angle—a sudden increase in artefact dimensions must be expected.

For such interventions, which necessitate a near orthogonal orientation to B_0_, it would be advantageous to diminish this effect by dynamically changing basic sequence parameters. The phase-encoding direction was taken into consideration for possibly having such a beneficial effect but showed only a minor influence in this work. This confirms existing results for metallic stents and needles in MRI—there was no significant effect of phase-encoding direction on artefact dimensions shown either [23, 25]. However, a notable exception was observed at the 90° position in the coronal plane, where switching the phase-encoding direction from AP to RL resulted in a substantial increase in artefact size. The underlying mechanism for this outlier remains unclear and may warrant further investigation.

This *in vitro* study has several limitations and exhibits future challenges that need to be discussed. Endovascular procedures are usually carried out under real-time image guidance; the static character of the presented experiment limits its generalisability in that regard. Although real-time compatible sequence parameters were used in this study, the balloon’s visualisation characteristics should be further evaluated under actual real-time imaging conditions in future work. The gadolinium-doped aqueous phantom model does not replicate the T1 or T2 relaxation properties of blood or soft tissue. Furthermore, blood flow and associated signal changes—depending on sequence type and parameters—might severely affect local imaging conditions. In clinical endovascular interventions, additional confounders not evaluated here may arise. For example, previously deployed metallic stents may produce susceptibility artefacts that interfere with the balloon’s visibility. Hence, further systematic testing is warranted in flow phantoms followed by animal studies. Additionally, bSSFP sequences at 3-T MRI are known to be prone to pronounced artefacts. Considering the increase in artefact dimensions with larger angle deviations relative to B_0_, it would be worthwhile to investigate the performance of emerging low-field MR scanners. Low-field MRI may exhibit a more stable artefact behaviour and could furthermore be advantageous in clinical use due to lower SAR levels. However, smaller susceptibility artefacts associated with lower field strengths may compromise the balloon’s conspicuity.

Another limitation of this proof-of-concept study is that only one measurement per condition was obtained. This was due to the limited availability of the specially produced prototype balloons. In particular, regarding the SPION concentration experiments, manufacturing variability cannot be excluded as a contributor to the nonlinear artefact behaviour. Further work should include repeated experiments with multiple balloons per condition.

Although the heating of SPION balloons has already been tested for MPI, potential thermal effects during MRI examinations should be evaluated. While acknowledging the different field topologies of MPI and MRI, and considering an existing study on a single stent type, the risk of heating of the balloon catheters in MRI is expected to be low [26]. Nevertheless, to support clinical translation, this issue should be investigated in a systematic and reliable manner, thereby also enabling an extensive statistical evaluation of the results.

The presented SPION-polymer balloon catheter design enables reliable device visualisation in 3-T MRI due to SPION-induced susceptibility artefacts under controlled phantom conditions. Beyond the investigated balloon, the general principle of SPION integration into instrument polymers may represent a promising approach to facilitate MRI-guided endovascular interventions. Further research under realistic physiological conditions, confirming these preliminary findings, is necessary to support this perspective.

## Supplementary information

ELECTRONIC SUPPLEMENTARY MATERIAL

## Data Availability

The datasets used and/or analysed during the current study are available from the corresponding author on reasonable request.

## Abbreviations

AP: Anterior-posterior
bSSFP: Balanced steady-state free precession
FA: Flip angle
FOV: Field of view
HF: Head-feet
ICC: Intraclass correlation coefficient
MPI: Magnetic particle imaging
MRI: Magnetic resonance imaging
RL: Right-left
SAR: Specific absorption rate
SNR: Signal-to-noise ratio
SPION: Superparamagnetic iron oxide nanoparticles
