# Supplementary material for: 3-T MRI in vitro characterisation of a SPION-polymer balloon catheter: a proof-of-concept study
Source: Eur Radiol Exp. 2026 Aug 13;10:114. doi: 10.1186/s41747-026-00778-z (PMC13473045; doi:10.1186/s41747-026-00778-z)
Supplement: Supplementary file 1 [file 41747_2026_778_MOESM1_ESM.pdf]

3-T MRI *in vitro* characterisation of a SPION-polymer balloon catheter: a proof-of-concept study

Electronic supplementary material

Supplementary Tables

Tab.1: Results of the manual artefact segmentation performed by two readers.

**Tested parameter**	**Artefact size (mm^2) – Rater A**	**Artefact size (mm^2) – Rater B**	**Artefact size (mm^2) –Mean**
5 wt% Balloon	261.84	244.75	253.30
20 wt% Balloon	234.99	193.48	214.23
30 wt% Balloon	342.41	278.32	310.36
Inflated 0°	239.26	198.36	218.81
Inflated coronal 30°	356.45	303.96	330.20
Inflated coronal 60°	501.10	429.08	465.09
Inflated coronal 90°	491.94	451.05	471.50
Inflated sagittal 30°	302.12	304.57	303.34
Inflated sagittal 60°	501.10	476.68	488.89
Inflated sagittal 90°	432.13	351.56	391.85
Deflated 0°	181.27	181.27	181.27
Deflated coronal 30°	373.54	329.59	351.56
Deflated coronal 60°	515.14	512.08	513.61
Deflated coronal 90°	669.56	593.26	631.41
Deflated sagittal 30°	416.87	397.34	407.10
Deflated sagittal 60°	692.75	481.57	587.16
Deflated sagittal 90°	629.27	578.00	603.64
FA 10°	259.40	256.35	257.87
FA 20°	241.09	219.73	230.41
FA 30°	216.67	159.30	187.99
FA 40°	236.21	186.16	211.18
FA 50°	232.54	191.04	211.79
FA 60°	223.39	198.97	211.18
PE 0° AP	217.90	187.99	202.94
PE 0° HF	240.48	194.09	217.29
PE coronal 90° AP	479.74	385.74	432.74

PE coronal 90° RL	598.75	559.69	579.22
PE sagittal 90° AP	443.12	372.92	408.02
PE sagittal 90° RL	462.65	398.56	430.60

Tab.2: Signal-to-Noise Ratio (SNR) values of the images acquired with different Flip angles (FA).

FA	SNR
FA 10°	4.07
FA 20°	7.98
FA 30°	10.54
FA 40°	13.59
FA 50°	14.07
FA 60°	16.82
